# Reversible Takotsubo Syndrome Complicated With Acute Thromboembolic Stroke Co-Occurring With Cola-Induced Severe Hypokalemia

**DOI:** 10.7759/cureus.16470

**Published:** 2021-07-18

**Authors:** Mohammed A Madkhali

**Affiliations:** 1 Internal Medicine, Jazan University Faculty of Medicine, Jizan, SAU; 2 Internal Medicine, The University of Toledo Medical Center, Toledo, USA

**Keywords:** cola-induced hypokalemia, takotsubo syndrome, hypokalemic paralysis, thromboembolic stroke, rhabdomyolysis.

## Abstract

Takotsubo syndrome (TTS) is a poorly understood phenomenon that manifests as reversible myocardial damage. Despite the overall benign course of TTS, acute complications and significant mortality and morbidity are frequently encountered. However, the risk factors for developing such complications are not well determined. We present a case of reversible TTS within 48 hours after correction of cola-induced hypokalemia complicated by thromboembolic stroke. We present the patient's clinical presentation with imaging studies and discuss the case in light of related literature. A 62-year-old woman with a longstanding history of inadequate oral intake and excessively sweetened cola soft drinks consumption. Presented with progressive symmetrical quadriparesis and found to have severe hypokalemia with rhabdomyolysis, elevated troponin, and acute left ventricular dysfunction. The patient was diagnosed with TTS and hypokalemic paralysis. Her hospital course was complicated with an acute thromboembolic stroke of bilateral occipital lobes and left cerebellum. Takotsubo cardiomyopathy was reversible 48 hours after correction of severe hypokalemia. Cardiac ischemic workup was negative for any evidence of myocardial ischemia. In this presented case, TTS was revisable within 48 hours after the correction of cola-induced hypokalemia. However, patients with similar presentations might have a higher risk for in-hospital thromboembolic complications of TTS.

## Introduction

Takotsubo syndrome (TTS) is a reversible myocardial damage that mimics the clinical manifestation of acute coronary artery disease (ACS). TTS is diagnosed in about 2% of patients presenting to the hospital with suspected ACS [1]. The precise pathophysiology of TTS is still unknown. However, multiple predisposing physical and mental risk factors have been proposed in retrospective studies to trigger TTS development [2]. Nevertheless, further longitudinal studies are required to prove the association between those risk factors and TTS. 

The overall natural history of TTS is benign; however, some of the affected patients developed acute complications with subsequent mortality and morbidity [3]. In this case, we present a patient with complicated clinical course consisting of TTS, hypokalemic paralysis, and rhabdomyolysis in the setting of cola-induced severe hypokalemia.

## Case presentation

A 62-year-old Caucasian woman presented to the emergency department with a history of progressive muscular weakness associated with nausea, vomiting, and diarrhea. At baseline, the patient was independently ambulating with a walker. However, she started to have a gradual bilateral upper and lower extremities weakness for two weeks before her presentation. As a result, she became dependent on her roommate to perform daily living activities (i.e., bathing, dressing, toileting, transferring, and feeding). She also reports an associated history of diarrhea three to four times a day since the onset of her symptoms. She reports a history of weight loss, poor appetite, polyuria, polydipsia, memory impairment, decreased visual acuity, and hearing loss on review of systems. She denied any history of shortness of breath or chest pain. The patient stated that she has been drinking 8-10 cans of 16 oz of Pepsi cola every day. The past medical history is remarkable for chronic obstructive pulmonary disease (COPD), history of abdominal trauma with subsequent splenectomy, gastroesophageal reflux with esophagitis and hiatal hernia status post failed Nissan fundoplication, alcohol abuse, and longstanding multiple psychiatric disorders treated with different antipsychotics and antidepressants. The patient stated that she had not taken any of her home medications for more than two weeks before the presentation. In addition, she reports a 20 pack-years history of cigarette smoking and lived with a roommate who has been busy lately and did not provide sufficient support to the patient. 

On physical examination, vital signs were normal; the patient looked cachexic and dehydrated with dry skin and mucous membranes. She was also confused with impaired remote memory and appeared in poor hygiene as she could not shower or sanitize her excreta for three to four days due to weakness. Neurological examination showed normal cranial nerves, a decreased strength in the upper and lower extremities (3/5 and 2/5, respectively), and normal sensation to soft touch. On abdominal examination, the patient was noted to have a midline surgical incision with reducible umbilical hernia, no abdominal tenderness was elicited, and she had normal bowel sounds.

**Figure 1:**
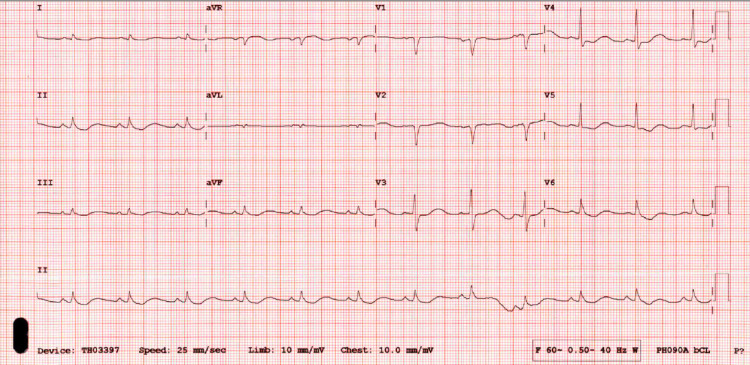
Initial EKG, serum potassium of 1.6 mmol/L.

Initial laboratory workup was remarkable for profound hypokalemia; serum potassium was 1.6 mmol/L (ref range: 3.5-5.0) with an elevated troponin of 2.63 ng/mL, brain natriuretic peptide (BNP) of 570 pg/mL, and total creatine kinase (CK) 1,391 U/L. Other electrolytes were normal, she had a serum sodium of 144 mmol/L, magnesium of 2.2 mg/dl, phosphorous of 2.7 mg/dl and calcium of 8.9 mg/dl. Furthermore, blood tests for complete blood count (CBC), creatinine, blood urea nitrogen (BUN), thyroid-stimulating hormone (TSH), free T4, lipid and coagulation profiles were unremarkable for any abnormalities. Liver function showed an elevated aspartate transaminase (AST) of 55 U/L (ref range: 0-41) but normal alanine aminotransferase (ALT) and alkaline phosphatase. Electrocardiogram on presentation showed a prolonged QT-interval (QTc 583 ms) with U-wave and ST-segment depression on lateral leads (Figure 1).

Therefore, an echocardiogram was done on the day of admission, and it showed a moderate to a severe reduction in the left ventricular ejection fraction (estimated LVEF of 30%-35%) associated with apical and anterior wall akinesia. It also showed moderate mitral and mild tricuspid valve regurgitations (Video 1). The patient was started on IV heparin infusion for presumed non-ST elevation myocardial infarction. 

**Video 1:** TTE on presentation showing reduced LVEF with apical and anterior walls akinesia. LVEF: left ventricular ejection fraction.

**Figure 2:**
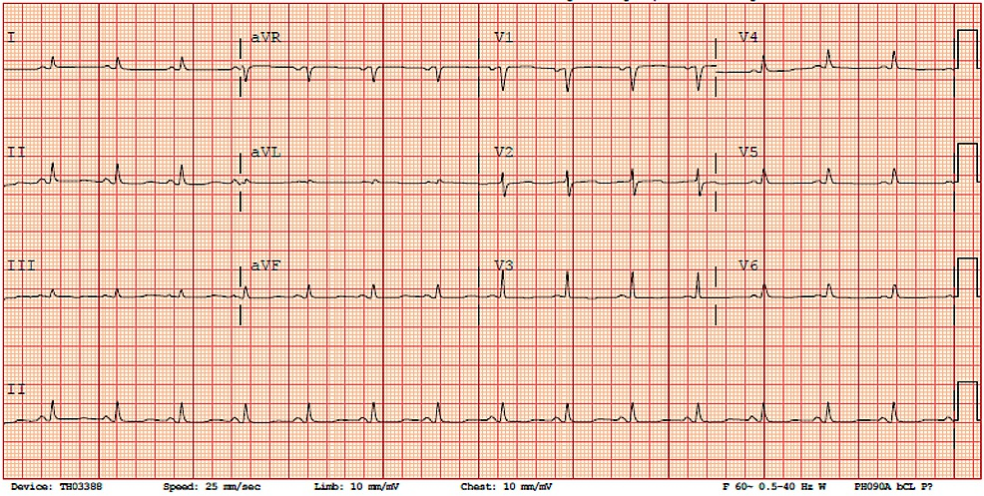
Follow-up EKG post correction of hypokalemia, serum potassium of 4.0 mmol/L.

Hypokalemia was corrected with intravenous and oral potassium chloride replacements. The serum potassium level returned to a normal value of 4.0 mmol/L on the third day of the hospital stay. After correcting hypokalemia, the follow-up EKG showed resolutions of ST-depression, improved QT-prolongation (QTc of 463 ms) and disappearance of U-wave (Figure 2). 

Further cardiac workup was negative for any evidence of myocardial ischemia on a nuclear stress test. Additionally, serum troponin level decreased to 0.53 ng/ml from 2.63 ng/ml on presentation. 

**Figure 3:**
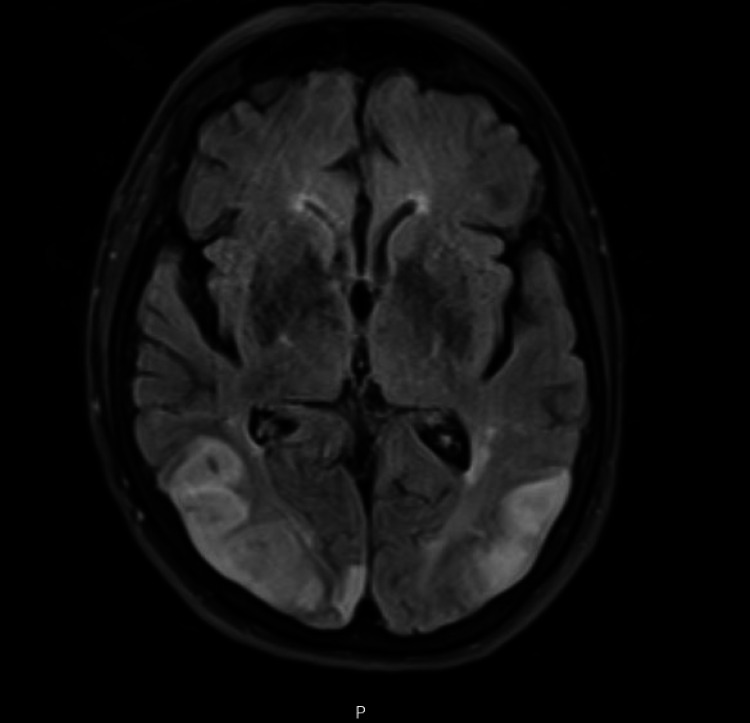
MRI brain showing diffuse abnormalities of bilateral posterior temporal lobes consistent with acute ischemia.

**Figure 4:**
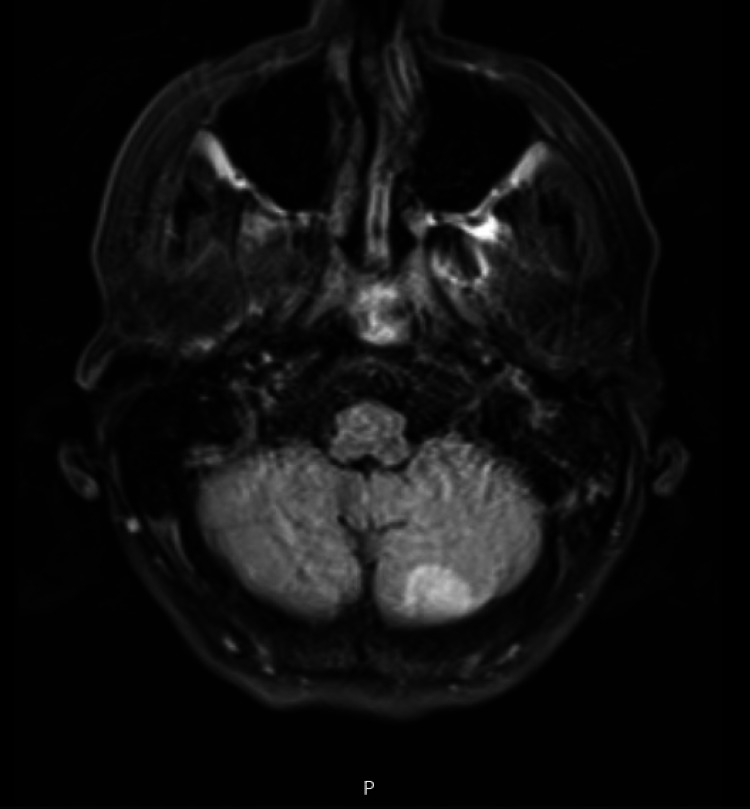
MRI brain showing left cerebellum diffusion abnormality consistent with acute ischemia.

Unfortunately, her hospital course was complicated with a sudden onset of bilateral visual loss, associated with decreased sensation on the left lower extremity and persistent weakness of bilateral upper and lower extremities. MRI brain showed diffusion abnormalities of bilateral posterior temporal lobes (Figure 3) and the left cerebellum (Figure 4) consistent with acute ischemia. 

Therefore, she underwent trans-esophageal echocardiogram to rule out left ventricular thrombus formation. It showed resolutions of left ventricular wall motion abnormalities with moderate to severe dilatation of the left atrium and no visualized thrombus in the left atrial appendage. Also, it showed an intact atrial septum, and no interatrial shunt on agitated saline bubble study (Video 2).

**Video 2:** TEE showing resolution of left ventricular wall motion abnormalities. TEE: transesophageal echocardiography.

Hence, the patient was diagnosed with reversible TTS associated with in-hospital complication of thromboembolic stroke. 

The patient was treated with anticoagulation and aspirin, IV heparin infusion was switched to oral rivaroxaban (xarelto) 20 mg daily, in addition to aspirin 324 mg daily for outpatient anticoagulation. On discharge, the serum level of potassium remained at a normal range with dietary replacement. She was discharged to a skilled nursing facility followed by cardiology and neurology clinics for further evaluation and management. 

## Discussion

This patient was presented with severe hypokalemia associated with asymptomatic left ventricular dysfunction and elevated cardiac markers suggestive of myocardial injury. Her complicated hospital course precludes the planned coronary arteries angiogram and LV angiography to rule out the obstructive coronary artery disease. Nevertheless, post potassium replacement, this patient had quick reversibility of cardiomyopathy as shown in the trans-esophageal echocardiogram with no evidence of ischemia on the nuclear stress test. Therefore, the other possibilities of myocarditis or coronary artery diseases (CAD) including vasospastic CAD are very unlikely in this case. Furthermore, this patient has multiple known risk factors for the development of TTS (i.e., female, postmenopausal, and history of psychiatric disorders) [2].

Low serum potassium is regarded as the most commonly encountered electrolyte abnormality in the clinical setting. Most patients with hypokalemia are found to have a mild to a moderate decrease of serum potassium below 3.6 mmol/L but more than 2.5 mmol/L. Less frequently, patients with severe hypokalemia (i.e., serum potassium of < 2.5 mmol/L) are predisposed to the risks of cardiac arrhythmias and muscle necrosis with subsequent rhabdomyolysis and ascending paralysis [4]. Hypokalemia can result from decreased potassium intake, extracellular to intracellular shift, and uncompensated gastrointestinal or renal loss of serum potassium. There are multiple reports of cola-induced severe hypokalemia complicated with myopathy, rhabdomyolysis, acute kidney injury, and atrial fibrillation following the excessive consumption of those drinks [5-8]. The proposed mechanism of cola-induced hypokalemia was discussed by Tsimihodimos et al.; they indicated that, when consumed excessively, the unabsorbed fructose content of cola soft drinks can cause osmotic diarrhea and subsequent gastrointestinal loss of potassium. Additionally, the high glucose content in those drinks could result in transient hyperinsulinemia and osmotic diuresis with a consequent intracellular shift of potassium and renal loss, respectively. Furthermore, the caffeine content of cola soft drinks could cause hypokalemia through intracellular potassium redistribution and its known diuretic effect [9]. In addition to excessive consumption of Pepsi cola drinks, this presented patient had other factors contributing to the severity of hypokalemia. She has been dependent upon sweeten soft drinks as a source of daily calories with decreased oral intake of potassium; additionally, the patient has chronic intermittent nausea with vomiting secondary to large hiatal hernia status post failed Nissan fundoplication. Apart from those factors, no other potential etiologies for hypokalemia could be considered in this case, including drug-induced hypokalemia since the patient has not been taking home medications - including proton pump inhibitor (PPI) and antipsychotics - for more than two weeks before her presentation. 

In a different clinical setting, severe hypokalemia has been proposed as a potential triggering factor for the development of TTS. For instance, TTS has been associated with severe hypokalemia secondary to primary aldosteronism and decreased oral intake [10,11]. Furthermore, excessive caffeine consumption from energy drinks and weight loss supplements has been linked to the occurrence of TTS [12,13]. However, to the best of our knowledge, there have been no reports of coexistent TTS with cola-induced severe hypokalemia. Therefore, the excessive consumption of caffeinated cola drinks in this patient could represent an unknown triggering factor for TTS. 

The prognosis of TTS is primarily benign in the majority of patients. However, there has been growing evidence highlighting the acute consequential mortality and morbidly associated with this disease. According to the international consensus review of TTS, the acute clinical course of TTS was complicated by atrial fibrillation in 5%-15% and LV thrombus formation in 2%-8% of the affected patients [3]. Nevertheless, the risk factors for developing those acute complications are still elusive. Cervellin et al. (2014) described an association between hypokalemia and elevated serum levels of D-dimer among patients with acute atrial fibrillation. Accordingly, this association could imply a higher risk of thromboembolism in the setting of hypokalemia [14]. The diagnosis of atrial fibrillation was neither evident during the presentation of our case nor detected through cardiac telemetry throughout her hospital stay. However, this does not preclude the possibility of paroxysmal atrial fibrillation with consequent LV thrombus formation before the patient’s presentation. As a matter of fact, in a recent retrospective study, the left ventricular thrombus was only detected in three out of 17 cases with ischemic stroke and TTS. Additionally, only 11% of patients with thromboembolic events were found to have in-hospital complication of atrial fibrillation. This study was conducted on 1,676 registered patients in the international Takotsubo database [15]. To sum up, the coexistence of TTS with cola-induced severe hypokalemia could carry a higher risk for major thromboembolic complications as described in this case.

## Conclusions

In this presented case, TTS was reversible within 48 hours after the correction of cola-induced hypokalemia. However, patients with similar presentations might have a higher risk for in-hospital thromboembolic complications of TTS. 
